# Improving understanding in the research informed consent process: a systematic review of 54 interventions tested in randomized control trials

**DOI:** 10.1186/1472-6939-14-28

**Published:** 2013-07-23

**Authors:** Adam Nishimura, Jantey Carey, Patricia J Erwin, Jon C Tilburt, M Hassan Murad, Jennifer B McCormick

**Affiliations:** 1 University of Washington, Seattle, WA, USA; 2 Mayo Clinic, 200 First St SW, Rochester, MN 55905, USA; 3 Department of Medicine, Mayo Clinic, Rochester, MN USA; 4 Knowledge and Evaluation Research Unit, Mayo Clinic, Rochester, MN USA; 5 Biomedical Ethics Research Unit, Mayo Clinic, Rochester, MN USA

**Keywords:** Informed consent, Comprehension, Systematic review

## Abstract

**Background:**

Obtaining informed consent is a cornerstone of biomedical research, yet participants comprehension of presented information is often low. The most effective interventions to improve understanding rates have not been identified.

**Purpose:**

To systematically analyze the random controlled trials testing interventions to research informed consent process. The primary outcome of interest was quantitative rates of participant understanding; secondary outcomes were rates of information retention, satisfaction, and accrual. Interventional categories included multimedia, enhanced consent documents, extended discussions, test/feedback quizzes, and miscellaneous methods.

**Methods:**

The search spanned from database inception through September 2010. It was run on Ovid MEDLINE, Ovid EMBASE, Ovid CINAHL, Ovid PsycInfo and Cochrane CENTRAL, ISI Web of Science and Scopus. Five reviewers working independently and in duplicate screened full abstract text to determine eligibility. We included only RCTs. 39 out of 1523 articles fulfilled review criteria (2.6%), with a total of 54 interventions. A data extraction form was created in Distiller, an online reference management system, through an iterative process. One author collected data on study design, population, demographics, intervention, and analytical technique.

**Results:**

Meta-analysis was possible on 22 interventions: multimedia, enhanced form, and extended discussion categories; all 54 interventions were assessed by review. Meta-analysis of multimedia approaches was associated with a non-significant increase in understanding scores (SMD 0.30, 95% CI, -0.23 to 0.84); enhanced consent form, with significant increase (SMD 1.73, 95% CI, 0.99 to 2.47); and extended discussion, with significant increase (SMD 0.53, 95% CI, 0.21 to 0.84). By review, 31% of multimedia interventions showed significant improvement in understanding; 41% for enhanced consent form; 50% for extended discussion; 33% for test/feedback; and 29% for miscellaneous.Multiple sources of variation existed between included studies: control processes, the presence of a human proctor, real vs. simulated protocol, and assessment formats.

**Conclusions:**

Enhanced consent forms and extended discussions were most effective in improving participant understanding. Interventions of all categories had no negative impact on participant satisfaction or study accrual. Identification of best practices for studies of informed consent interventions would aid future systematic comparisons.

## Background

Obtaining informed consent is a key component of biomedical research that seeks to uphold the ethical value of patient and participant autonomy. Yet, the process has become increasingly burdensome for both researchers and research participants. Obtaining consent is by no means simple: researchers must not only enroll participants in their study, but also convey to them their rights as human subjects, the scientific question under investigation, the study methodology, and thepotential harms and benefits. Research staff exert considerable effort documenting that process.

Research consent typically emphasizes disclosure on the presumption that more information aids potential participants in decision-making [1]. A review of the clinical research informed consent literature from 1961 to 2006 revealed that only 54% of patients and research participants adequately understood the aim of a given study; 50% understood the process of randomization; 47% understood the nature of voluntariness; 44% understood the ability to withdraw; 50% understood the risks of the study; and 57% understood the benefits of a study [2]. Given the premise that the understanding of information improves participants’ decision-making capacity, these results offer a humbling glimpse at what “informed” currently entails.

In a landscape of expanding biomedical research methods—such as genomic sequencing, data sharing, bio-banking, creating pluripotent stem cells, and linking medical record data with genomic sequence—the need for effective and efficient consent processes has become paramount. Since the mid-1990s the National Institutes of Health (NIH) have invested significant resources on research studies on the informed consent process. Recommendations on how to modify existing consent templates have come from a number of scholars and working groups [3-9]. Moreover, in July 2011 the Office for Human Research Protections (OHRP) issued an Advanced Notice of Proposed Rulemaking (ANPR) to Human Subjects Research Oversight that proposed several important changes to informed consent under certain conditions.Most notably, the ANRP called for “…greater specificity about how consent forms should be written and what information they should contain.” Researchers and their institutions, they suggested, should “[create] forms that are shorter, more readily understood, less confusing, that contain all of the key information, and that can serve as an excellent aid to help someone make a good decision about whether to participate in a study” [10]. The ANPR may prompt further quality improvement studies about best practices.

In recent decades bioethics investigators, Institutional Review Boards, and funders have invested in improving the informed consent process. These improvements or enhancements often rely on altering standard paper consent documents or elements of the process, or medium in which information is delivered or by which consent is obtained. For example investigators have tested enhanced design methodologies [2], multimedia tools and technologies [11], altered counseling or dialogue [12], and the provision of quizzes that give immediate feedback [13]. Many of these efforts aim to shorten the consent form document and increasing potential participants’ understanding of what was explained to them.

A recent systematic review could not determine whether several important consent innovations—those involving multimedia, extended discussions or immediate feedback quizzes, had an impact on understanding [14]. However, small numbers prohibited a full meta-analysis and direct comparison was not possible due to heterogeneity in study methodologies and assessment techniques.

Here we report a systematic review of randomized controlled trials of informed consent interventions for biomedical research and an accompanying meta-analysis.

## Methods

This systematic review and meta-analysis are consistent with existing reporting guidelines (the PRISMA statement) [15].

### Eligibility criteria

**Figure 1:**
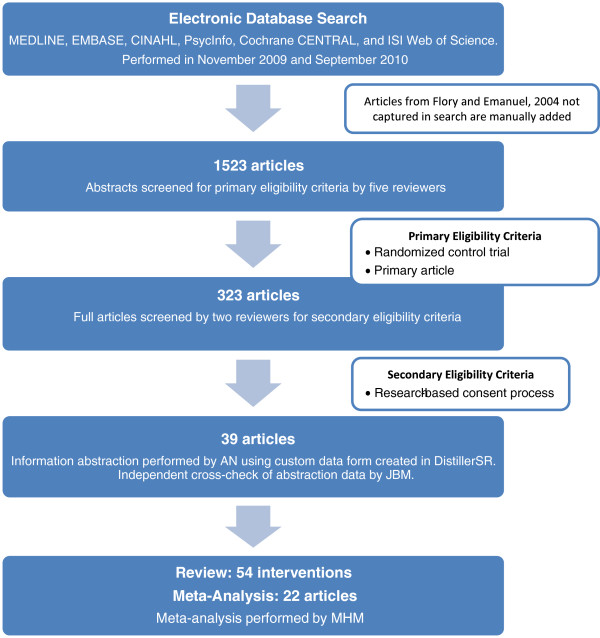
Flow diagram of results from search process for articles.

We included randomized controlled trials (RCT) that evaluated interventions designed to improve the informed consent process for patients and/or research participants. Studies simulating informed consent process were also included, if they were RCT. We excluded all case reports, single cohort studies, non-randomized trials, review articles, and other systematic reviews (Figure 1).

### Search strategy

An expert reference librarian designed and conducted an electronic search strategy with input from study investigators. The search spanned from database inception through September 2010. The initial search was run on Ovid MEDLINE using a combination of medical subject headings (MeSH) and text-words, and then translated into the terms appropriate to Ovid EMBASE, Ovid CINAHL, Ovid PsycInfo and Cochrane CENTRAL. The ISI Web of Science and Scopus databases were searched as well, using text-words. The primary subject headings used were “informed consent” or “consent”. The strategy further focused on formats in which the information might be delivered: “video recording”, “audiovisual aid”, “computer-assisted instruction”, “multimedia”, or “patient education materials”. Outcome and study types were the final strategy filters: comprehension, readability, pre-post testing, research design, decision making, patient satisfaction, clinical trials. We cross-referenced our results with the bibliography of the most recent and comprehensive systematic review on the topic published in 2004 [14].

### Assessment of study eligibility

Four reviewers working independently and in duplicate screened all abstracts and titles and, upon retrieval of candidate studies, three team members (JCT, JBM, AN) reviewed the full text to determine eligibility. If the study was eligible, data were abstracted by AN. Any questions arising during data abstraction were resolved by discussion with other team members. JBM conducted an independent cross-check.

### Data extraction and synthesis

Through an iterative process, we created a standardized form to extract descriptive, methodological and key variables from all eligible studies. Distiller (Ottawa, Ontario), an online reference management system for systematic reviews, was used to manage study selection and data extraction. We collected data on study design, population, demographics, intervention, and analytical technique.

Data were tabulated and categorized according to the type of intervention. We used categories similar to those published previously: *multimedia, enhanced consent form*, *extended discussion*, *test/feedback*, and *miscellaneous methods*[14].

### Primary outcome: understanding

Our primary outcome was participant understanding/knowledge. Understanding/knowledge refers to a participant’s objective comprehension of information conveyed in the informed consent process, as assessed by quantitative testing methods. Measurements of participants’ perceived levels of understanding were not included in this study. We further divided understanding into immediate knowledge, knowledge retention, and reading time.For the purposes of this analysis we defined immediate knowledge as that tested within one week of the control or intervention procedure.

### Secondary outcomes: satisfaction and accrual

Data regarding the impact of interventions on participant satisfaction levels or accrual rates were also tracked and synthesized. Satisfaction rates were determined in certain studies by quantitative surveys or interviews. Rates of accrual were tracked based on the actual enrollment of participants into the parent studies associated with an informed consent intervention. Only actual accrual rates were synthesized in our results. A sufficient number of actual studies were deemed available to draw conclusions on accrual.

### Other key variables

We also evaluated whether a study had an actual, parent study with which it was associated; whether the control and intervention of a study were proctored, or overseen, by a member of the study team, for example a study nurse, an investigator, or a research assistant; the sample size of a trial; the demographics of the study population; and the methods by which outcomes were assessed. In addition, we determined whether or not a study had a “standard control” versus an “enhanced control”. As control techniques varied between studies, we used the classification of standard control to delineate those studies that compared their interventions to a basic, minimally-altered consent form required by their institutions’ IRB. If a study designated its non-intervention as either “standard,” “industry standard,” or “legally-required,” we considered it the control.

### Meta-analysis of understanding scores

Due to the difference in the methods of outcome ascertainment (understanding score), we estimated from each study the standardized mean difference (SMD) and 95% confidence interval. The SMD is an effect size measure that is unit-less and can be pooled across studies that evaluate a similar outcome assessed with different scales. In all analyses, SMD was estimated as the intervention understanding scores minus the control understanding scores (i.e., SMD > 0 favors the intervention group). Random-effects meta-analysis was used to pool SMDs across studies because heterogeneity of the subjects, knowledge content and the design of the studies was anticipated [16]. Random-effects modeling is a conservative approach that combines within-study and between-study variance. We estimated heterogeneity statistically using the I-squared statistic, which represents the proportion of true heterogeneity that is not attributable to chance [17]. The reasons for heterogeneity were explored by conducting a test of interaction based on whether the intervention group had a proctor, the control group had a proctor, whether the control group was an enhanced form of the consent form or just a standard form, the setting of the study (real vs. simulated) and the type of subjects (healthy volunteers vs. patients) [18]. To avoid double counting the control groups of studies with multiple intervention arms, we tested in sensitivity analyses the inclusion of each arm and determined if changing the intervention group affected study conclusions.

## Results

### Search results

Study selection process is described in Figure 1. In sum, 39 primary articles were reviewed. As some of these articles tested more than one intervention, we ultimately tracked the results of 54 interventions: sixteen multimedia (Table 1), twenty-two enhanced form (Table 2), six extended discussion (Table 2), three tests/feedback (Table 3), and seven miscellaneous (Table 4) interventions were reviewed. In addition to the interventions identified in the 2004 systematic review [14], we were able to identify 24 additional studies fitting our criteria: six multimedia, nine enhanced consent forms, four extended discussions, three tests/feedback, and five miscellaneous.

**Table 1:** Results of trials of video and computer multimedia interventions

	**Standard control**				**Sample size**	***Understanding scores, %***		
**Source**		**Intervention**	**Population**	**Scenario**		**Control**	**Intervention**	**P Value**
Karunaratne et al., 2010	Y	Interactive computer presentation replaces consent form. Graphics, video clips, links, and text styling	Patients with DM	Simulated	60	**73**	**82**	0.005
Kass et al., 2009 (formerly Agre et al. 2003)	N	Digital touch-screen presentation on oncology clinical research replaces brochure	Patients with cancer	Real	130	17	34	0.03‡
Bickmore et al., 2009	Y°	Standard consent form with explanation by interactive, computerized “agent”	Healthy volunteers	Simulated	18	39	42	NS
Hack et al., 2007	N°	Supplementary take-home audiotape recording of standardized study details°	Patients with cancer	Real	42	88	85	NS∞
	N°	Supplementary take-home audiotape recording of IC consultation and second audiotape of standardized study details°	Patients with cancer	Real	47	88	87	NS∞
Hutchinson et al., 2007	Y°	Supplementary 10 min. video. Vignettes, visual aids, voice-over, and graphics. Patients allowed to take video home°	Patients with cancer	Real	173	NA	NA	0.011§,∞
Mittal et al., 2007	N°	Powerpoint slideshow on computer replaces consent form. Graphics, text styling, summaries video and embedded voice narration°	Patients with cognitive impairment or Alzheimer's disease	Simulated	35	52	60	NS
Wirshing et al., 2005 (formerly Agre et al. 2003)	N	Video with specific study guidelines replaces generic video on human research. Professionally acted vignettes, bulleted text, and audio narration	Patients with mental illness and healthy volunteers	Simulated	261	69	73	< 0.0001
**Campbell et al., 2004**	**Y**	**Video replaces consent form. Voicenarration and no reading required**	**Parents of pediatric research participants**	**Simulated**	**120**	**47**	**50**	**NS**
	**Y**	**Computer presentation replaces consent form. Video and still pictures; bulleted text with audio narration**	**Parents of pediatric research participants**	**Simulated**	**118**	**47**	**51**	**NS†**
**Agre and Rapkin, 2003**	**N**	**Computer presentation replaces consent form. Summary sections (?)**	**Patients with cancer and healthy volunteers**	**Real**	**209**	**68**	**66**	**NS**
	**N**	**Video replaces consent form. Professionally produced with summary sections**	**Patients with cancer and healthy volunteers**	**Real**	**221**	**68**	**73**	**NS**
**Dunn et al., 2003 (includes Dunn et al., 2001)**	**Y°**	**Powerpoint slideshow replaces consent form. Altered organization, layout and summaries of text. Narrated by study staff°**	**Patients with mental illness and healthy volunteers**	**Real**	**99**	**85**	**91**	**0.014**
**Fureman et al., 1997**	**N°**	**Supplementary 26 min. video. Talkshow format with expert panel answering audience questions°**	**Injection drug users**	**Real**	**186**	**81**	**80**	**NS**
**Weston et al., 1997**	**N°**	**Supplementary, professionally filmed 10 min. video°**	**Pregnant women**	**Simulated**	**90**	**91**	**95**	**NS**
**Llewellyn- Thomas et al., 1995**	**Y**	**Standard consent form in navigable, digital format**	**Patients with cancer**	**Simulated**	**100**	**81**	**79**	**NS**

Abbreviations: NA not Available; NS Not significant; DM diabetes mellitus.

Bolded rows indicate trials include in the 2004 systematic review by Flory and Emanuel.

° Human proctor available for question/answer.

§ P-value is a based on change in pre-consent and post-consent quiz scores.

†P-value is a post hoc calculation of the total scores contrasting all combinations of formats; Understanding scores based on averages of high and low-risk protocol arms.

‡ Only relates to participant’s ability to identify purpose of the study.

∞ Understanding assessed at approximately 1 week.

**Table 2:** Results of trials with enhanced consent form intervensions

	**Standard control**				**Sample size**	***Understanding scores, %***		
**Source**		**Intervention**	**Population**	**Scenario**		**Control**	**Intervention**	**P Value**
Paris et al., 2010	Y	Simplified paper document with systematic readability improvement	Patients with stroke, DM, or OSAS	Simulated	115	67	69	NS
	Y	Simplified paper document developed by a working group of clinical research nurse, IRB member, and healthy volunteer	Patients with stroke, DM, or OSAS	Simulated	114	67	69	NS
Campbell et al., 2008	N	Simplified text in booklet format with color. 7th grade reading level	Healthy volunteers	Simulated	146	64	85	<0.001
Walters and Hamrell, 2008	Y	Simplified paper document. 6th grade reading level	Healthy volunteers	Simulated	317	77	79	NS*
Paris et al., 2007	Y	Simplified paper document with systematic readability improvement	Healthy volunteers	Simulated	99	78	82	≤0.05
	Y	Simplified paper document developed by a working group of clinical research nurse, IRB member, and healthy volunteer	Healthy volunteers	Simulated	101	78	83	≤0.017
	Y	Simplified paper document developed by a working group and by systematic readability improvement	Healthy volunteers	Simulated	100	78	82	≤0.05
**Campbell et al., 2004**	**Y**	**Simplified paper document with revised layout, text styling, and added pictures**	**Parents of pediatric patients**	**Simulated**	**119**	**47**	**53**	**NS**
**Agre and Rapkin, 2003**	**N**	**Simplified paper document presented in booklet form with summary section. 8th grade reading level**	**Patients with cancer and healthy volunteers**	**Real**	**221**	**69**	**70**	**NS**
**Coyne et al., 2003**	**Y°**	**Simplified paper document with revised text styling, page layout, and language. 7th grade reading level. Organized in Q/A format°**	**Patients with cancer**	**Real**	**207**	**69**	**72**	**NS‡**
**Dresden and Levitt, 2001**	**N**	**Simplified paper document with revised layout, text styling, and language**	**Patients in the E.R.**	**Simulated**	**100**	**72**	**88**	**<0.0001**
**Stiles et al., 2001**	**Y°**	**Simplified paper document with text styling and graphics°**	**Patients with mental illness and healthy volunteers**	**Simulated**	**227**	**81**	**81**	**NS**
**Bjorn et al., 1999**	**N**	**Leaflet used by pharmacology company with revised language, style and layout. Hypertension scenario**	**Healthy volunteers**	**Simulated**	**135**	**48**	**56**	**<.05**
	**N**	**Leaflet used by pharmacology company with revised language, style and layout. Sterilization scenario**	**Healthy volunteers**	**Simulated**	**100**	**NA**	**NA**	**NS**
**Murphy et al., 1999**	**N**	**Simplified paper document. 6th grade reading level with text styling and illustrations**	**Women at risk for HIV**	**Simulated**	**141**	**70**	**83**	**0.0001**
**Davis et al., 1998**	**Y**	**Revised with patient input, readability improved from college to 7th grade level, shortened, booklet format, graphics**	**Patients and healthy volunteers**	**Simulated**	**183**	**56**	**58**	**NS**
**Rogers et al., 1998**	**Y°**	**Consent required to “opt out” of study, rather than “opt in”°**	**Recent mothers**	**Real**	**44**	**30**	**47**	**<0.02**
**Taub et al., 1987**	**Y**	**Simplified paper document. 7th grade reading level**	**Elderly volunteers**	**Real**	**235**	**68**	**70**	**NS**
	**N**	**Standard or simplified paper document with “Letter Gothic”, enlarged font**	**Elderly volunteers**	**Real**	**235**	**71**	**65**	NS*
	**N**	**Standard or simplified paper document with “Orator”, enlarged all-caps font**	**Elderly volunteers**	**Real**	**235**	**71**	**70**	**NS**
**Taub et al., 1986**	**Y**	**Simplified paper document. 7th grade reading level**	**Patients with heart disease**	**Real**	**188**	**71**	**74**	**NS**
**Epstein and Lasagna, 1969**	**Y**	**Simplified paper document. Shorter with succinct phrasing**	**Hospital Employees**	**Simulated**	**44**	**45**	**67**	**<.001**

Abbreviations: *NA* Not Available, *NS* Not Significant, *DM* diabetes mellitus, *OSAS* obstructive sleep apnea syndrome, *HIV* human immunodeficienc virus.

Bolded rows indicate trials included in the 2004 systematic review by Flory and Emanuel.

°Human proctor available for question/answer.

* Significant improvement reported for eldest cohorts.

‡ Understanding assessed within approximately 1 week.

**Table 3:** Results of trials of extended discussion intervensions

	**Standard control**				**Sample size**	***Understanding scores, %***		
**Source**		**Intervention**	**Population**	**Scenario**		**Control**	**Intervention**	**P Value**
Sarkar et al., 2010	Y°	Standardized, focus group discussion with study nurse in place of individual counseling°	Parents of malnourished children in Vellore, India	Real	118	73	73	NS
Freer et al., 2009	Y	Information leaflet and supplementary, standardized discussion with study staff°	Parents in United Kingdom	Simulated	21	69	85	0.015
	Y	Information leaflet and supplementary, standardized discussion with study staff°	Parents in United States	Simulated	20	66	75	NS
**Aaronson et al., 1996**	**Y°**	**Same presentation formats. Supplementary telephone conversation with oncology nurse°**	**Patients with cancer**	**Real**	**230**	**66**	**83**	**<.001§**
**Tindall et al., 1994**	**Y**	**Supplementary conversation with enrolling physician°**	**Patients with HIV**	**Real**	**113**	**60**	**63**	**NS∞**
Simes et al., 1986	Y°	Uniform consent procedure ensuring total disclosure of information by physicians°	Patients with cancer	Real	57	62	74	0.0001*

Abbreviations: *NA* Not Available, *NS* Not Significant, *HIV* human immunodeficiency virus.

Bolded rows indicate trials included in the 2004 systematic review by Flory and Emanuel.

°Human proctor available for question/answer.

* P value is for the overall outcomes which include "willingness to participate" and "anxiety".

§ Scores based on an “awareness scale” in an interview format after approximately 1 week.

∞ Understanding assessed at approximately 1 week.

**Table 4:** Results of trials of test/feedback interventions

**Source**	**Standard control**				**Sample size**	**Understanding scores,%**		
		**Intervention**	**Population**	**Scenario**		**Control**	**Intervention**	**P Value**
Eyler et al., 2004	Y	Supplementary, scripted Q/A by study staff throughout narration. Staff provided correct answer immediately after posing question^°^	Patients with mental illness	Real	24	57	68	NS
	Y	Supplementary, scripted Q/A by study staff throughout narration. Staff provided correct answer after patient answered a question^°^	Patients with mental illness	Real	32	57	67	NS
Taub et al.,1983	N^°^	Participant asked to repeat knowledge quiz (up to three times) if any questions were answered incorrectly^°^	Elderly volunteers	Real	100	69	89	<.01

Abbreviations: *NA* Not available, *NS* Not significant, *Q/A* Question and answer.

°Human proctor available for question/answer.

Results were updated from four multimedia interventions that were previously unpublished and assessed from a secondary source [19-22]. In the enhanced form category, two interventions applying different font styles for the elderly were added. These interventions were present in a 1987 study by Taub et al. but not in the 2004 review. Another enhanced consent form trial was updated from a prior secondary source [21]. A test/feedback intervention was dropped; though it was previously deemed subject to a randomized trial, it was only tested on participants who did not score perfectly on the first iteration of an assessment quiz [23]. Conversely, an additional trial, categorized as longitudinal in the 2004 review, but reassessed by us as randomized control, was added to the test/feedback category [24]. One trial, previously present in the miscellaneous category, was excluded from our primary outcome calculations as its results were concerned with long-term retention, rather than immediate understanding [25].

**Figure 2:**
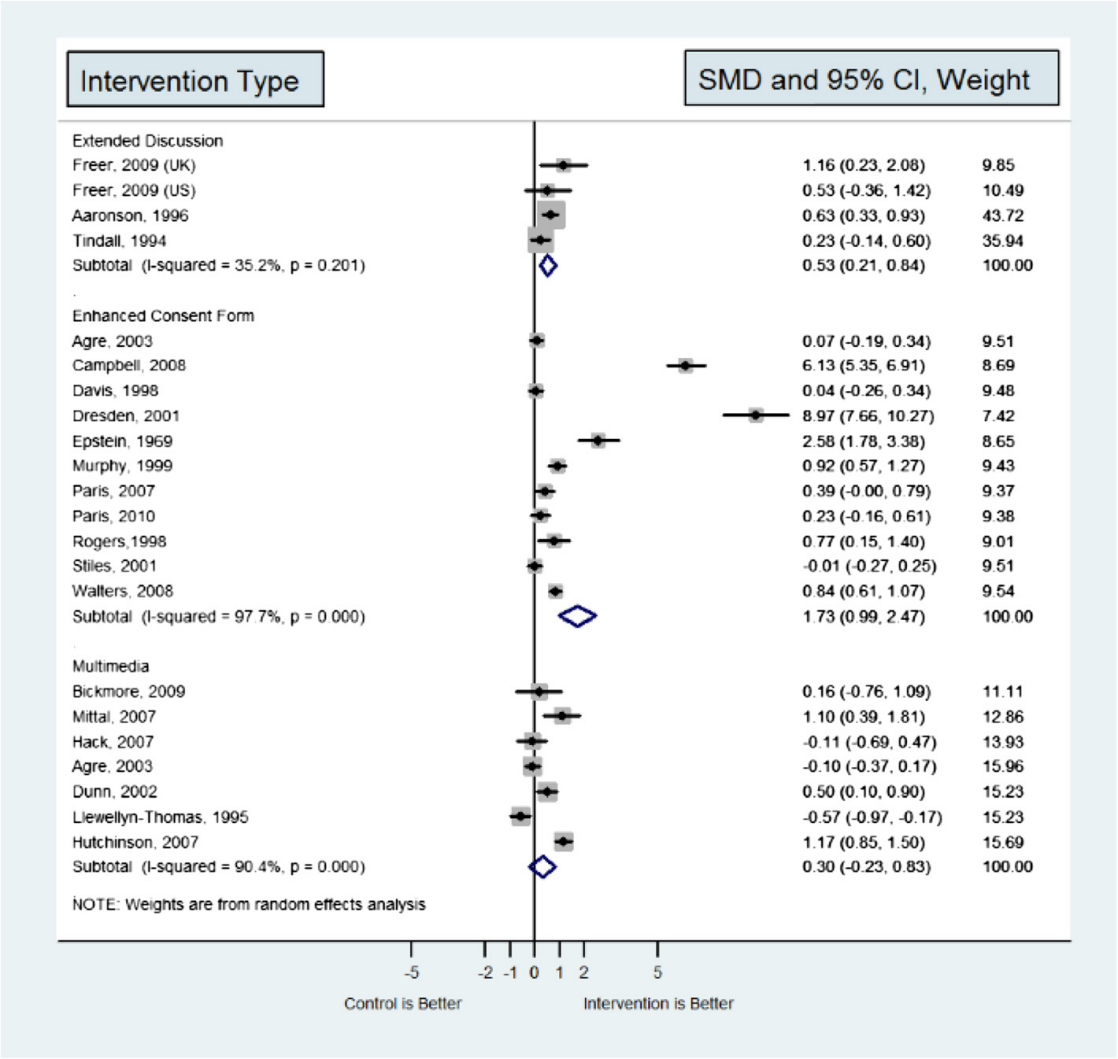
Meta-analysis of interventions reviewed.

Meta-analysis was feasible with seven multimedia, eleven enhanced form, and four extended discussion studies (Figure 2). Remaining studies from all five categories did not provide their understanding assessment data in a format that could be included in the meta-analysis.

### Traits of the studies

Most of the studies that had a human proctor present for the control group also had one present for the intervention group. There was one exception in the multimedia category [19], and several in the extended discussion or test/feedback categories [20-22]. Less than half of the multimedia (44%) and enhanced consent (14%) trials had a human proctor present for the administration of either informed consent procedure. 57% of the miscellaneous interventions had a proctor, while the enhanced discussion and test/feedback interventions all had human proctors.

Forty-four percent of the interventions were tested under real, as opposed to simulated, conditions. All test/feedback interventions had real parent studies, whereas only seven enhanced form interventions were in real scenarios (31%). All newly tested enhanced form interventions since 2004 were in simulated trials. Multimedia, extended, and miscellaneous studies were split in their extent of being tested under real conditions (50%, 50%, and 40%, respectively).

Assessment formats varied widely between studies, including in-person or telephone questionnaires [23-30], or computer- and paper-based quizzes. Certain studies tested in an open-book format [19], allowing participants access to trial information, while most studies required participants to work from memory alone. The number of questions within a test ranged from 8 to 80, with question types including True/False, Yes/No, multiple choice, open-ended, and prompted recall. As seen in Tables 1, 2, 3, 4 and 5, some studies had mean understanding scores for both control and intervention below 50%, while others had these scores both in the upper 80th percentile. Such variation suggests a potential difference in assessment difficulty between trials. While some assessment methods were validated for their respective study populations through pilot methods, validation for the population under study was not a consistent feature for all methods.

**Table 5:** Results of trials of mixed and miscellaneous intervensions

	**Standard control**				**Sample size**	***Understanding scores, %***		
**Source**		**Intervention**	**Population**	**Scenario**		**Control**	**Intervention**	**P Value**
Tait et al., 2010	N	Online presentation in which tables, instead of text, are used to explain risk vs benefit	Parents	Simulated	3139	49∞	45∞	NS
	N	Online presentation in which pictographs, instead of text are used explain risk vs benefit	Parents	Simulated	3094	49∞	67∞	<.05
Ford et al., 2008	Y°	Standard paper document read aloud by study staff with familymember or care-giver present°	Elderly patients with Parkinson's disease	Real	136	48	52	0.012‡
Lavori et al., 2007	Y°	Supplementary self-assessment for study staff after each consent discussion with a participant°	Patients and healthy volunteers	Real	836	78	79	NS
**Agre et al., 2003 (Merz et al. trial)**	**Y°**	**Supplementary educational vignettes°**	**Patients undergoing apheresis and healthy volunteers**	**Simulated**	**206**	**NA**	**NA**	**NS**
**Stiles et al., 2001**	**N°**	**Neutral facilitator present at research participant's meeting with investigator°**	**Patients with mental illness and healthy volunteers**	**Simulated**	**227**	**82**	**81**	**NS**
Wragg et al., 2000	N	Simplified paper document and video with physician explanation. Material written to express current facts without stressing importance of the trial	Female research participants	Simulated	100	51	49	NS

Abbreviations: *NA* Not Available, *NS* Not Significant.

Bolded rows indicate trials included in the 2004 systematic review by Flory and Emanuel.

°Human proctor available for question/answer.

‡ Understanding assessed at approximately 1 week.

∞Scores represent the percent of sample who scored >5 out of 7, which they called adequate knowledge.

### Impact of different consent innovations on understanding

#### Multimedia

Meta-analysis of multimedia-based consent approaches showed a non-significant increase in understanding scores compared with control consent approaches (SMD 0.32, 95% CI, -0.20 to 0.85). Heterogeneity of this analysis was substantial (I^2^ = 90%). Results are depicted in Figure 2. Subgroup analyses (Table 6) were not statistically significant and did not provide an explanation of the observed substantial heterogeneity. Due to this heterogeneity, the meta-analysis results should be tempered with contextual data from the component studies.

**Table 6:** Subgroup interactions for meta-analysis of interventions

**Multimedia vs. control**					
	**# of studies**	**SMD**	**LL**	**UL**	**p-value**
**Control**					
Nonstandard	3	0.23	−0.41	0.88	0.8
Standard	4	0.37	−0.45	1.19	
Overall	7	0.29	−0.22	0.8	
**Proctor present for control?**					
No	1	0.57	0.17	0.97	0.65
Yes	5	0.37	-0.44	1.17	
**Proctor present for intervention?**					
No	2	0.51	0.14	0.88	0.85
Yes	4	0.41	-0.52	1.34	
**Subjects**					
Patients	4	0.69	0.14	1.25	0.33
Volunteers	1	0.16	-0.76	1.09	
**Setting**					
Real	4	0.12	-0.65	0.89	0.25
Simulated	3	0.64	0.22	1.06	
**Enhanced consent form vs. control**					
	**# of studies**	**SMD**	**LL**	**UL**	**p-value**
**Control**					
Nonstandard	3	2.33	0.01	4.66	0.49
Standard	8	1.47	0.7	2.23	
**Proctor present in both groups?**					
No	7	2.6	1.33	3.88	0.01
Yes	2	0.33	-0.43	1.09	
**Subjects**					
Patients	4	2.56	0.69	4.44	0.59
Volunteers	5	1.94	0.66	3.23	
**Setting**					
Real	3	0.57	-0.06	1.19	0.01
Simulated	8	2.23	1.19	3.28	
**Extended discussion vs. control**					
	**# of studies**	**SMD**	**LL**	**UL**	**p-value**
**Proctor present in control group?**					
No	3	1.31	0.33	2.29	0.19
Yes	1	0.63	0.33	0.93	
**Subjects**					
Patients	2	1.33	-0.07	2.74	0.53
Volunteers	2	0.83	0.19	1.47	
**Setting**					
Real	1	2.06	1.61	2.52	0.01
Simulated	3	0.66	0.39	0.93	

Abbreviations: *SMD* Standard mean deviation, *LL* Lower limit, *UL* Upper limit.

Several contextual details of the control procedures and intervention design of the studies included in the meta-analysis are of note. Three of the included trials had non-standard controls. Mittal et al. sought to test the feasibility of two interventions (multimedia and enhanced consent form) for a larger trial [31]. They specified no control, but the data allowed us to assign the enhanced form as a non-standard control. Hack et al. compared two types of audiotape supplements, but neither were compared to a tapeless scenario [32]. Agre and Rapkin compared several interventions to a consent form that was modified to read at the 8th grade level [33]. Relatedly, Bickmore et al. tested an interactive computer-based proctor against two different procedures: the participant read an informed consent document alone or the participant was led through consent process by a human proctor. We included the data comparing the computer-based proctor (intervention) to the human proctor (control), which showed no significant difference. Yet, both the computer and human proctors were comparably more effective than the read-alone scenario for patients with adequate health literacy (p = 0.006) [19]. Finally, as noted elsewhere [34], the multimedia intervention tested by Llewellyn-Thomas et al. in 1995 was merely a standard consent form presented on a computer screen, representing variability in what the category of “multimedia” entails [35].

Six additional studies were reviewed but not included in the meta-analysis due to the absence of requisite data in their published results. Fureman et al. and Weston et al. had non-significant results for immediate understanding, though their interventions improved retention rates [36,37]. While the intervention of Kass et al. significantly improved understanding rates, our review relied on only one question from the assessment: the ability of a participant to identify the purpose of the study [23]. Campbell et al. tested an intervention in two different theoretical trials, one involving a high-risk protocol; the other low-risk [26]. We averaged the results for both, which were similar and non-significant. Finally, Wirshing et al.’s study found strong demographic predictors in intervention efficacy [38]. The team compared a specialized video to a general IRB video with university students, Veterans Affairs (VA) patients, and patients with schizophrenia. While all showed significant improvement in understanding post-intervention, the students benefited the most. Results from other studies included data for populations with mental illnesses [31,38,39], history of injecting drug use (IDU) [36], and varying stress levels [33].

Based on the entire review, and not just meta-analysis, five of the sixteen total multimedia interventions (31%) resulted in significant improvement in understanding.

### Enhanced consent form

Meta-analysis showed that the enhanced consent form category was associated with significant increase in understanding scores compared with control consent approach (SMD 1.73, 95% CI, 0.99 to 2.47). Heterogeneity of this analysis was substantial (I^2^ = 97%). Results are depicted in Figure 2. Subgroup analyses (Table 6) demonstrated that the improvement in understanding scores was larger in simulation studies (vs. real-setting studies) and in studies in which neither study arm (intervention or control) had a human proctor. These subgroup interactions (simulated and human proctor) partially explain the observed heterogeneity.

In addition to the impact of simulation and presence of a proctor, other factors may have impacted study heterogeneity in the meta-analysis. One variation included the risk factors and medical literacy of a study’s sample demographic. Epstein and Lasagna’s study population was composed primarily of medical employees [40]. Three other studies included populations that had mental illness [41], low literacy levels [27], or low-income levels [42]. Another source of heterogeneity is the way in which researchers assessed participant understanding. Unlike the paper-based testing formats used by most studies, Rogers et al. used an interview-based assessment technique [43]. Though the impact of this technique is unclear, it is correlated with markedly lower scores for both control and intervention cohorts than in other studies (Table 2).

Of the studies not included in the meta-analysis, only one had significant results. Bjorn et al. used a leaflet intervention for two different study scenarios. For the scenario involving a theoretical hypertension study, the results were significant, but for a sterilization scenario, results were non-significant [44]. Campbell et al., Agre and Rapkin, Coyne et al., Davis et al. and Taub et al. all had non-significant results. However, Agre and Rapkin, as with their trial of a multimedia intervention, used a control form with improved readability potentially decreasing the perceived effectiveness of the intervention [33]. Murphy et al. saw significant improvement with their intervention, but only for a specific population of low-income women at-risk for HIV [42] Taub et al. focused primarily on an elderly population, finding little significant improvement except for when an enlarged font was used for the eldest cohort [45].

Overall, nine out of twenty-two enhanced consent interventions (41%) showed significant improvement in the review.

### Extended discussion, test/feedback, and miscellaneous

The approach of using extended discussion was associated with significant increase in understanding scores compared with control consent approach (SMD 1.03, 95% CI, 0.79 to 1.26). Heterogeneity of this analysis was minimal (I^2^ = 35%). Results are depicted in Figure 2. Subgroup analyses (Table 6) demonstrated that the improvement in understanding scores was larger in real-setting studies (vs. simulation studies). These subgroup interactions partially explain the minimally observed heterogeneity.

Studies not included in the meta-analysis for this category of intervention showed varied results. Sarkar et al. tested a focus group intervention with parents in rural India, finding no significant improvement [13]. Aaronson et al., conversely, found significant improvement with extra phone conversations [29]. Both studies assessed outcomes with an interview-style format. Simes et al. reported positive results after providing a check-list to ensure physicians had a comprehensive disclosure discussion with trial patients [46]. For all studies, in both meta-analysis and review, three of six (50%) extended discussion interventions had a significantly positive impact on understanding.

Of the two new test/feedback interventions, neither showed significant improvement in understanding [22]. However, an intervention reassessed in this review as part of a randomized control trial did show a significantly positive effect [24]. None of the other interventions from the 2004 review were randomized, thus they were not included in this analysis. In total, one-third of the test/feedback interventions included in our review resulted in significant improvement.

Two of the five new miscellaneous interventions showed significant improvement in understanding [47,48]. While Flory and Emanuel presented one of three randomized trials with significant improvement (33%) [14], we observed improvement in two out of seven total trials (29%).

### Sensitivity analysis

Four studies in the meta-analysis had multiple intervention arms [32,49-51]. The choice of intervention arm did not affect the overall conclusions of this meta-analysis.

### Impact of different consent innovations on satisfaction, accrual and retention

There is little to no indication that the interventions tested in these trials negatively impact participant satisfaction or study accrual. Data regarding rates of participant satisfaction were tracked for eleven interventions. Preferences between interventions and controls were either insignificant or showed that participants significantly preferred the interventions [19,27,29,32,35,43,47,52-54]. Accrual rates, tracked for trials with real parent studies, were also reported as improved or unchanged by interventions in all categories [13,23,29,43,52,55,56], with one exception [46]. These findings are consistent with those discussed in the 2004 review [14].

Ten studies that tested an intervention’s impact on understanding also tested for impact on knowledge retention; interventions in all categories except extended discussion and test/feedback showed an overall positive impact on rates of knowledge retention. The seven interventions in the multimedia and enhanced form categories significantly improved knowledge retention rates [25,36,37,50]. Three interventions in the other categories had insignificant results for retention [24,46,48].

An additional five studies tested seven different interventions’ impact on retention, but not immediate understanding. Of these studies, all found significantly positive impacts from their interventions. These studies included three enhanced forms [53], one test/feedback [57], and three miscellaneous interventions [55,58,59].

## Discussion

Different types of intervention to improve research consent across a variety of settings show mixed impact on improving participant knowledge. The search and data abstraction for this paper adds twenty-four interventions, tested in randomized control trials, to Flory and Emanuel’s 2004 systematic review. In addition, this first of its kind meta-analysis, notwithstanding the stated limitations, demonstrates that enhanced consent forms, extended discussion, and multimedia interventions favor improved knowledge outcomes. Only enhanced consent forms and extended discussion interventions were significant in changing the outcome; the consistency of multimedia’s effectiveness remains unclear. Finally, there is little evidence that a participant’s satisfaction or a study’s accrual rates would be negatively altered by attempts to improve the informed consent process, which should be reassuring to investigators. Our findings confirm those from other previously performed systematic reviews. However, they may not be applicable to illiterate or socially disadvantage groups, given the limits of our search, indicating an important domain for more research.

Our data highlight several key points. First, both the form and the conversation are important for the process.Secondly, the effect of multimedia in the informed consent process remains in question. However, as found in the 2004 review, it may prove useful in helping with long-term knowledge retention [14]. This element of learning may promote other dimensions of participant autonomy such as voluntariness: if a participant better remembers what a study is about, he or she can better decide whether to stay enrolled. Moreover, we speculate that multimedia may serve to compliment discussion and written forms to simultaneously address immediate and long-term understanding. Defining the role of multimedia will be increasingly critical as media-based technology becomes cheaper to implement, more accessible and more transportable (e.g., iPads).

Still, even the most exquisitely-designed form, be it on paper or computer screen, should not be expected to suffice. Arguably there is still no substitute for a good conversation, which facilitates opportunities for questions and interaction cannot be underestimated. An interesting and novel focus for improving consent could be creating interventions designed explicitly on improving communication skills Our data suggest that if the research enterprise could move towards a practice of rich conversations between investigator and participant, several crucial points would be addressed. Participants may have a better understanding of the study, they would likely feel more like a “partner” in the research process, they could be easily exposed to a natural test/feedback setting, and a relationship between the participant and the researcher would be established. We found that extended discussions had the most consistent result of improving understanding, although only 50% showed significant improvement. No category of intervention managed to have consistent efficacy.

The question of whether “shorter forms are better than longer forms” – at least whether “shorter is no worse than longer” for participant understanding – is still an open question, pointing to the need for studies that directly compare a “short form” intervention to a traditional “long form” control in a randomized control setting. Whether short forms coupled with conversation would potentiate even greater increases in understanding is a hypothesis for future consent research.

The efficacy of test/feedback interventions is difficult to discern at this point. Most test/feedback trials fall easily into the format of a pseudo-randomized or cohort study as *only* the participants who fail a first assessment proceed to take a second (or third) assessment. Researchers must devise more stringent methodologies to ensure that their studies are fully legitimate randomized experiments.

### Limitations

Our study has several limitations. We restricted our search to interventions tested in RCT trials in order to meet the general gold standard of a systematic review. Yet, others have suggested that certain complex, socially-embedded biomedical activities are restricted by the lens of an RCT [60]. The informed consent process may be such an activity and may find ample aid from other more qualitative or observational studies. In addition, our study is limited by the primary outcome we sought to investigate: understanding. We were focused on objective assessments of participant understanding. Rates of understanding are important under the presumption that more information aids potential participants in decision-making [1]. Information-based decision-making aims to demonstrate respect for individual participants’ autonomy.However, there are other ethical values that may be equally, if not more, important to participants. For example, the values of trust and honesty may be more significant to participants who are willing to enter a trial based on their physicians’ recommendations. Truly informed consent may thus entail more than simply comprehending and memorizing information.

Several features of our analysis reveal the need for standardized research on informed consent interventions. The controls used in the studies we analyzed were highly varied. In some studies the control consent process was similar to standard practice. Usually, this process involved a long, paper-based document at the 12th grade (or higher) reading level [56]. In other studies, the control was an already enhanced version of the standard form. For example, Mittal et al.’s control form was a well-designed, formatted brochure revised to read at the 8th grade reading level [31]. One would expect the improvements in understanding—*over the control*—for trials with enhanced control formats to be less significant than those with standard control formats. In some cases, the control cohort did not have any form, likely amplifying the results of the intervention [25]. Thus, side-by-side comparison of these different interventions may be unfair.

Heterogeneity across these trials was the norm for assessing understanding. The wide ranges in normalized scores for both control and intervention between studies suggest that the varied choice of approach across studies may contribute to perceived level of understanding. While all trials included are interested in a participant’s level of understanding after the consent process, the different testing formats produced varied results. Assessments differed drastically in format, question-type, length, and provision and this heterogeneity among assessments hinders direct comparison. Standardized, verified examinations have been developed specifically for the informed consent process, yet only several studies in our review made use of them. For example, the MacCAT-CR, BICEP and QuIC are all pre-established assessment tools that were used in several studies [19,31,48]. However, the majority of studies used custom-created assessment forms. While a number of teams determined the reliability of their tests through pilot versions or other means, the inconsistency between different trials still precludes an accurate comparison. Just as the composition of an informed consent document may impact understanding levels, so too may assessment methods impact the degree to which participants can express their knowledge. This issue is particularly relevant for populations with low-literacy levels. Comparing results between trials with strongly divergent assessment techniques is problematic and limits the value of systematic reviews.

Additional variations between studies that limit comparison have been discussed by previous authors. The categories with the most positive outcomes, enhanced discussion and test-feedback, were also the categories with the fewest number of studies [14]. Few adequate criteria exist for tracking study quality; there are vast differences between population demographics; and categories for interventions group disparate interventions together. In 2009, Henry et al. focused in depth on each study in Flory and Emanuel’s multimedia category, exposing how almost every study could be interpreted differently than it was in the systematic review. They agreed with the authors of a Cochrane review of multimedia informed consent interventions: “the empirical literature is not yet sufficiently developed to draw definitive conclusions one way or the other about the general effectiveness of or value derived from multimedia consent aids” [11,61]. We agree with this conclusion of Henry et al. and Ryan et al. and further extended its implications to include all of the intervention categories. Perhaps more important than our findings for improving the consent process are our findings for improving the *study* of the consent process.

Despite these limitations and potential theoretical flaws, our research contributes an important body of data to the study of understanding in the informed consent process.

## Conclusions

We report on a systematic review of 54 interventions and meta-analysis of 22 interventions that sought to improve the rates of participant understanding in the informed consent process. The attention to research informed consent in recent years highlights the need for up-to-date, concise summaries of effective consent strategies. However, such summaries have thus far remained elusive. Furthermore, in an area of increased attention to consent for genomic research and bio-banking, knowing the state of the literature on effective consent strategies is essential. In an attempt address these needs, we report a systematic review of randomized controlled trials of informed consent interventions for biomedical research and an accompanying meta-analysis.

The findings of our study suggest that enhanced consent forms and extended discussions are most effective in improving participant understanding. Multimedia interventions are effective, though not significantly so, and appear to be more useful for improving long-term knowledge retention rates. Interventions involving test/feedback quizzes may be effective, but studies are too sparse to currently draw useful conclusions. Miscellaneous interventions do not appear to consistently improve understanding, though studies of such interventions are also sparse.

For many of the people who do research with human participant, obtaining informed consent is viewed as a legal and ethical hurdle that stands in the way of the real study of interest. Guidelines for research informed consent need to be cost-effective for researchers and institutions to implement. The most accessible route to providing useful, applicable evidence on better research informed consent process to researchers obtaining consent is by standardizing how studies on informed consent are conducted. Such standardization (or streamlining) need not diminish or eliminate creativity with interventions developed to enhance the informed consent process, but rather should be done in a way that will allow for comparisons of interventions in meta-analysis and systematic reviews.

## Competing interests

The authors declare that they have no competing interests.

## Authors’ contributions

AN, JBM, and JCT screened abstracts. AN, JCT, and JBM abstracted data. JBM, JCT, and MHM designed the study. PJE designed and conducted the search. MHM performedthe meta-analysis.AN and JBM drafted the manuscript. All authors reviewed and edited as necessary the manuscript and approved the final manuscript.

## Pre-publication history

The pre-publication history for this paper can be accessed here:

http://www.biomedcentral.com/1472-6939/14/28/prepub
